# Can health information through mobile phones close the divide in health behaviours among the marginalised? An equity analysis of Kilkari in Madhya Pradesh, India

**DOI:** 10.1136/bmjgh-2021-005512

**Published:** 2021-07-26

**Authors:** Diwakar Mohan, Kerry Scott, Neha Shah, Jean Juste Harrisson Bashingwa, Arpita Chakraborty, Osama Ummer, Anna Godfrey, Priyanka Dutt, Sara Chamberlain, Amnesty Elizabeth LeFevre

**Affiliations:** 1 Department of International Health, Johns Hopkins University Bloomberg School of Public Health, Baltimore, Maryland, USA; 2 Computational Biology Division, Department of Integrative Biomedical Sciences, Institute of Infectious Disease and Molecular Medicine (IDM), Faculty of Health Sciences, Observatory, Western Cape, South Africa; 3 BBC Media Action, New Delhi, Delhi, India; 4 Oxford Policy Management, New Delhi, Delhi, India; 5 Research and Policy, BBC Action Media, London, UK; 6 School of Public Health and Family Medicine, University of Cape Town, Cape Town, South Africa

**Keywords:** Epidemiology, Maternal health, Public Health

## Abstract

Kilkari is one of the largest maternal mobile messaging programmes in the world. It makes weekly prerecorded calls to new and expectant mothers and their families from the fourth month of pregnancy until 1-year post partum. The programme delivers reproductive, maternal, neonatal and child health information directly to subscribers’ phones. However, little is known about the reach of Kilkari among different subgroups in the population, or the differentiated benefits of the programme among these subgroups. In this analysis, we assess differentials in eligibility, enrolment, reach, exposure and impact across well-known proxies of socioeconomic position—that is, education, caste and wealth. Data are drawn from a randomised controlled trial (RCT) in Madhya Pradesh, India, including call data records from Kilkari subscribers in the RCT intervention arm, and the National Family Health Survey-4, 2015. The analysis identifies that disparities in household phone ownership and women’s access to phones create inequities in the population eligible to receive Kilkari, and that among enrolled Kilkari subscribers, marginalised caste groups and those without education are under-represented. An analysis of who is left behind by such interventions and how to reach those groups through alternative communication channels and platforms should be undertaken at the intervention design phase to set reasonable expectations of impact. Results suggest that exposure to Kilkari has improved levels of some health behaviours across marginalised groups but has not completely closed pre-existing gaps in indicators such as wealth and education.

**Summary box**

- By necessity, ‘direct to beneficiary’ mobile health programmes target those with phone access and thus exclude the poorest and most marginalised.
- Understanding inequities in who participates and benefits from mobile health programmes is vital for designing strategies to reach the most marginalised.
- Exposure to Kilkari appears to benefit the higher educated among the poorest in terms of wealth quintiles.
- Therefore, education may be a key enabler to help improve health behaviours among those exposed to Kilkari content.
- The way forward for digital ‘direct to beneficiary’ communication programmes may be to customise and target health information based on the characteristics of specific segments of the population.

## Introduction

There is much interest in ‘direct to beneficiary’ digital health communications programmes as a potential mechanism for addressing critical gaps in access to and receipt of health information, particularly in low-income and middle-income countries.^1–10^ Maternal mobile messaging programmes are among the few examples of such programmes that have scaled widely in different settings.^11 12^ Yet, to reach target women with health information, such programmes usually require beneficiaries to have access to a mobile phone as a prerequisite for participation. While overall rates of phone ownership are increasing globally, disparities in household ownership, and, more specifically, women’s access to and use of phones, persist.^13–15^ In India, despite near universal phone access among men, only half of women have access to mobile phones.^16^ Women with access to phones have poorer quality phones then men and frequently find their use constrained by a range of factors from phone sharing practices to social norms around securing credit.^17 18^

Kilkari is the one of the largest maternal mobile messaging programmes in the world, available to subscribers free of charge. Established in 2013 in Bihar, India, Kilkari had scaled to 13 states to reach over 10 million subscribers by April 2019. Subscribers received up to 90 min of content via up to 72 weekly calls from the fourth month of pregnancy until the child is 1 year old.

Understanding inequities in who participates and benefits through the life of a programme is vital for designing strategies to reach the most marginalised, and to optimise impact. Despite emerging evidence linking maternal messaging programmes to changes in health outcomes, little is known about the differential effects of these programmes across key population subgroups, including the least educated, poorest and most ethnically disadvantaged.^7 12 19–23^

In table 1, we present a framework for assessing equity in ‘direct to beneficiary’ health programmes by adapting the Tanahashi healthcare access framework and applying it to Kilkari in India.^24^ Data were drawn from three sources: an endline survey of women participants who received the Kilkari intervention as part of a randomised controlled trial (RCT) in Madhya Pradesh, call data records from Kilkari subscribers in the RCT sample and data on household mobile phone ownership from the Indian National Family Health Survey (NFHS-4) India from 2017 to 2020.^25 26^ A detailed description of the data sources and analytical methods are provided in box 1.

Box 1 **Description of data sources and analytical methods**

As part of efforts to determine the impact of Kilkari on key reproductive maternal newborn and child health behaviours, an individually randomised controlled trial (RCT) was conducted in four districts of Madhya Pradesh, India from 2017 to 2020.^25 26^ This analysis explores programme eligibility, enrolment, reach, exposure and affect across three dimensions of equity—education, caste and wealth. The eligibility and enrolment were based on comparisons to the National Family Health survey-4 data which surveyed households and women of reproductive age all across India. The questions considered for the analysis include whether a household lists a mobile phone in its assets (household ownership) and if women report having a mobile phone they can use (women’s access to mobile phones). The health outcomes (affect) considered in this analysis is based on self-reports at endline by women in the trial who received the intervention. The outcomes included standard indicators from the maternal and child continuum of care such place of childbirth, infant feeding and postpartum contraceptive practices. The subscribers’ reach and exposure to the intervention was assessed using call records stored by the programme database with data on the status (answered or not) and the duration of the call. Years of schooling completed was used to create four categories based on educational level—no schooling (0 years), primary (1–5 years), secondary (6–10 years) and higher (10 or more years). Caste categories were based on the classification established by the Government of India—that is, scheduled castes, schedule tribes, other backward classes and the general caste category. Wealth categories were based on dividing the survey sample into quintiles using a score created by a principal components analysis of household assets.^37^ Survey analyses were carried out with R V.3.4 and graphics generated with ggplot2 package.^38^ Summary measures of equity were produced using the WHO HEAT PLUS application, designed to help equity analysis using disaggregated estimates from various data sources.^39–41^ To assess inequities in health outcomes among those enrolled, we looked at subscribers exposed and not exposed to Kilkari in the intervention arm of the RCT using two summary measures of equity^1^: Slope of Index Inequality (SII) and^2^ Relative Concentration Index (RCI). The SII is an absolute weighted measure of inequity which provides an overall estimate of changes in health behaviour across the dimensions of equity assessed—wealth, caste and education in our case. Bounded between the values of −100 and +100 (if multiplied by 100), the SII indicates the absolute difference in estimated values of an indicator between the most-advantaged and most-disadvantaged using a regression model. In contrast, the RCI is a relative measure which reflects proportional inequality and thus an understanding of where one group stands in relation to other groups.^41 42^ Both SII and RCI take the value zero if there is no inequality with higher values indicating greater inequality. Positive values indicate a concentration among the advantaged, while negative values indicate a concentration among the disadvantaged.^41 43^

**Table 1:** Mapping the concepts of reach and coverage of digital programmes to the Tanahashi framework

Components of study framework	Definition	Application to Kilkari
Who benefits from the type of information provided by programmes like Kilkari?	Population for whom the changes in health outcomes are needed	All pregnant and postpartum women and their husbands in India
Who is eligible to receive Kilkari?	Target population who meets inclusion criteria and can potentially participate	All pregnant and postpartum women with reported access to a mobile phone who are beyond the twelfth week of pregnancy or have an infant less than a year old
Who is subscribed (enrolled) to Kilkari?	Population subscribed to the programme	All pregnant and postpartum women registered by front-line workers in government tracking databases with functional phone numbers, who are beyond the twelfth week of pregnancy or have an infant less than a year old
Who is reached by Kilkari?	Population receiving one or more components of the programme	Subscribers who have answered at least one call (a ‘successful’ call)
Who is exposed to Kilkari content?	Population receiving minimum threshold of programme to cover core components	Subscribers who have listened to at least 50% of the cumulative content of all Kilkari calls that they are eligible to receive
Who is affected by Kilkari?	Population receiving programme that reports changes in target behaviours	Subscribers to Kilkari who reported changes in target RMNCH behaviours

RMNCH, reproductive, maternal, newborn and child health.

### Who benefits from the type of information provided by programmes like Kilkari?

In theory, stage appropriate reproductive, maternal, neonatal and child health (RMNCH) information is expected to benefit all households with pregnant women and new mothers with infants under 1 year of age. Across the 13 states where Kilkari implementation is ongoing, an estimated 50 million women become pregnant every year.^27^ Data on the sociodemographic characteristics of those who have a ‘need for information’ among this population are limited. However, 2015 NFHS data indicate that total fertility rates are highest among the poorest and among those with no schooling, which may suggest greater representation of marginalised groups among pregnant and postpartum women than in the broader population at any given time.^28^ While it is difficult to quantify which subgroups have the most ‘need for information’, it would not be unreasonable to assume that the less educated, poorest and most marginalised castes are likely to benefit from such an intervention.^29–31^

### Who is eligible to receive Kilkari?

To be eligible to receive Kilkari in 13 Indian states, women must be pregnant and beyond the twelfth week of gestation, or have an infant less than a year old and, most importantly, must be able to provide their health worker with a mobile phone number (personal or otherwise) to receive prerecorded weekly calls. There are significant differences in household level ownership of mobile phones across key sociodemographic characteristics, and these inequities are more pronounced for women’s reported access to mobile phones.^16^ Data from 2015 NFHS indicates that the gap in household mobile phone ownership between the advantaged general caste (GC) and least advantaged scheduled tribe (ST) is 15.4%, while the gap in women’s access to mobile phones between the GC and ST is 27.1%.^16^ These differences in household ownership of phones and women’s reported phone access were different in magnitude between the poorest and richest (21% and 47%) segments of the population, and those with higher education and no schooling (13.9% and 55.3%).^16^ Overall differentials across these sociodemographic characteristics in women’s phone access contributed to the introduction of inequities in the population eligible to receive Kilkari. These inequalities need to be considered by programme implementers designing digital ‘direct to beneficiary’ health communication interventions so that reasonable expectations of programme reach, and impact can be set, and to prevent the exacerbation of inequities.

Kilkari was originally designed as part of an integrated social and behavioural change communication programme in the state of Bihar to strengthen RMNCH practices and generate demand for public health services. Communication was layered through different channels, including face-to-face communication by front-line health workers, to increase reach and exposure and improve diffusion of information.^32^ While several interventions were adopted by other state governments, and nationally, they were scaled independently, according to different timelines, which rarely overlapped. Other interventions were not scaled at all, creating gaps in the programme’s theory of change.^33^

### Who is subscribed (enrolled) to Kilkari?

**Figure 1:**
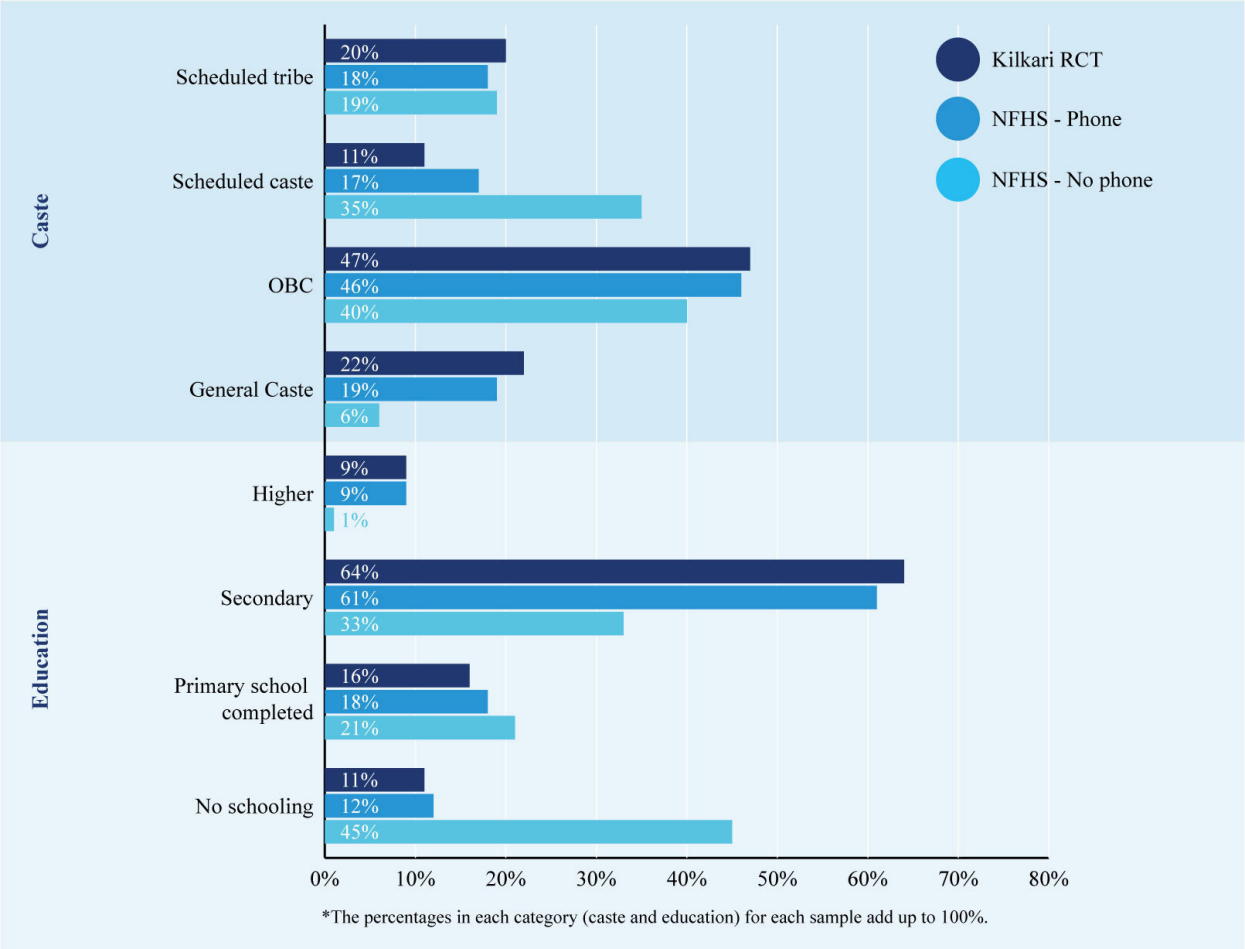
Caste and education of enrolled participants in the intervention arm of the Kilkari randomised controlled trial (RCT) as compared with their distribution among women reporting phone access from National Family Health Survey (NFHS-4). OBC, other backward classes.

While the sociodemographic profile of those subscribed to Kilkari at scale is not available, the RCT sample in Madhya Pradesh, where respondents were enrolled directly at the community level by study researchers, provides information on characteristics such as wealth, caste and education.^25^ Figure 1 depicts differences in the distribution of key characteristics for women with and without phone access in rural Madhya Pradesh based on the NFHS population level survey, as compared with the women who were enrolled in the intervention arm of the Kilkari RCT, who reported having access to a phone between the hours of 7:00 and 20:00. The restricted time criteria were required to comply with the Telecommunications Regulatory Authority of India’s directive that automated prerecorded outbound calls must be made within socially acceptable hours. A comparison of the NFHS and RCT samples (figure 1) conveys two key points^1^: the RCT sample is similar in profile to the population-based NHFS sample of women 15–49 years of age with access to a phone (denoted by the grey vs red bars), and^2^ there are stark differences in educational levels and caste between those women enrolled in the Kilkari RCT and those without access to a mobile phone according to the NFHS survey (grey vs blue bars). These differences underscore the higher educational level of those enrolled in even the most basic of ‘direct to beneficiary’ mobile health communication programmes—that is, programmes that can be accessed from the most basic mobile phone with no additional software or skill beyond answering a phone call.

## Who is reached by Kilkari?

Once enrolled in Kilkari, subscribers can receive up to 72 weeks of calls starting in the fourth month of pregnancy until the child is a year old. Calls are timed to match the gestational age at enrollment and therefore, the number of calls per subscriber may vary in accordance with the timing of entry into the programme. Subscribers are ‘reached’ if the Kilkari call is ‘successful’ —that is, the call not only reaches the handset but is answered. To optimise reach, Kilkari attempts to call the same subscribed mobile phone numbers up to nine times each week—three times the first day, and two times over the next 3 days—to reach a target subscriber. Subscribers are given the option to unsubscribe from the service at any time. Although there are no significant differences in ‘successful’ call rates by caste, those with no schooling and the poorest are the least likely to answer at least one Kilkari call. One caveat is that a successful call pick up does not guarantee that the messages were picked up by the target subscriber, especially in large rural households, phone calls are picked up by children or other family members.

**Figure 2:**
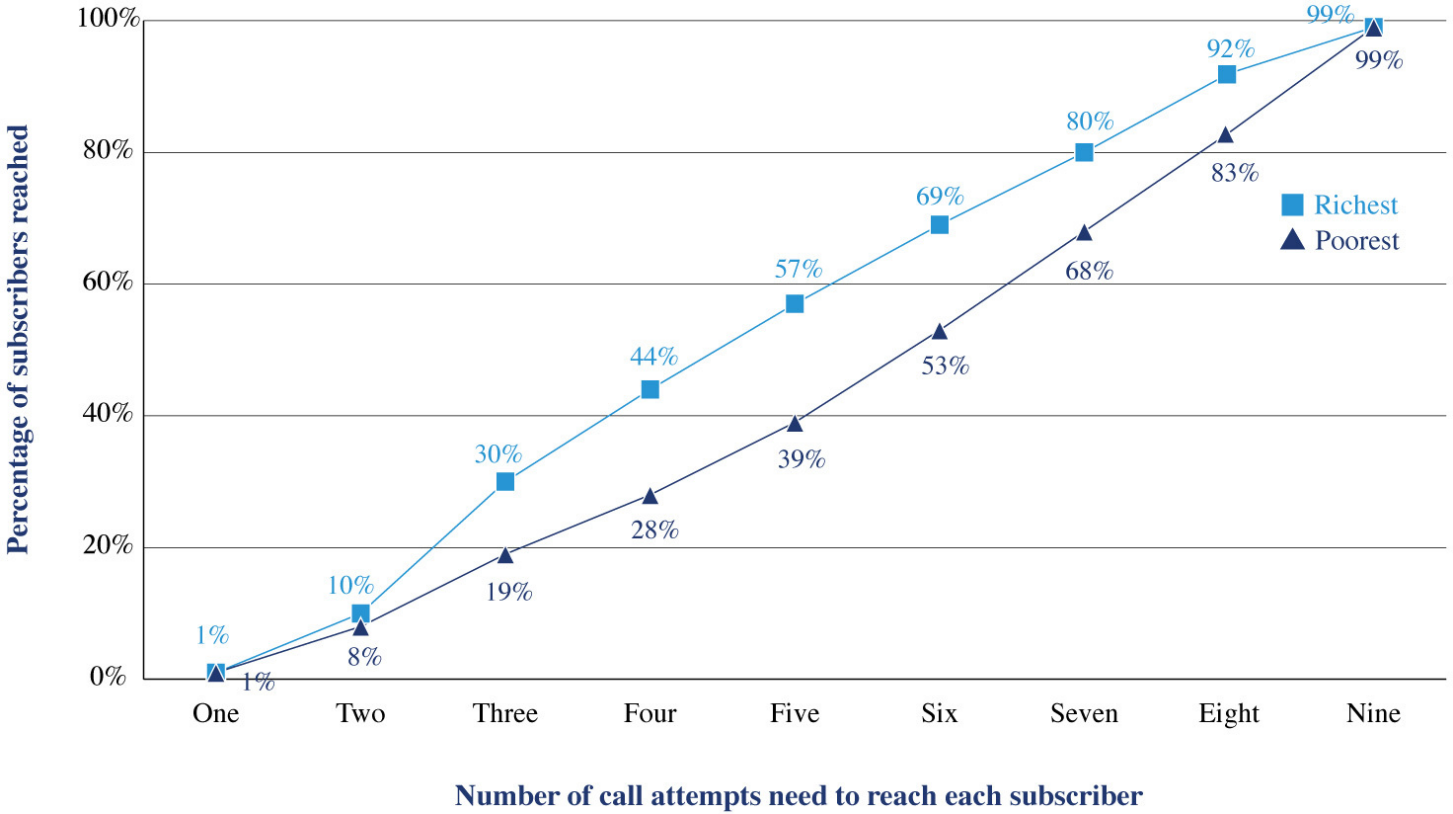
Number of call attempts made to successful reach a Kilkari subscriber for the richest and poorest quintiles of subscribers in the intervention arm of RCT. RCT, randomised controlled trial.

Figure 2 illustrates the number of call attempts it takes to reach a subscriber among the richest and poorest 20% of the RCT intervention arm sample, by wealth. On average, 60% of subscribers from the richest quintile have been reached by the fifth call attempt as compared only 40% of those from the poorest quintile. The number of attempts needed to successfully reach a subscriber may vary based on a range of factors including sociodemographic characteristics, phone characteristics (available credit, functionality), environmental factors (available electricity, location of top-up shops) and the social norms underpinning phone access and use.

### Who is exposed to Kilkari content?

Kilkari calls range from 60 to 108 s in length. Calls are comprised of three parts: introduction to the topic (20% of the length of an individual call), core content (60%) and closing (20%). In this study, to be considered ‘exposed’, subscribers needed to listen to 50% or more of the cumulative content they were eligible to receive. This definition is different from that used in the impact paper, which instead considered exposure to specific content about specific health practices to assess causality.^25^

**Figure 3:**
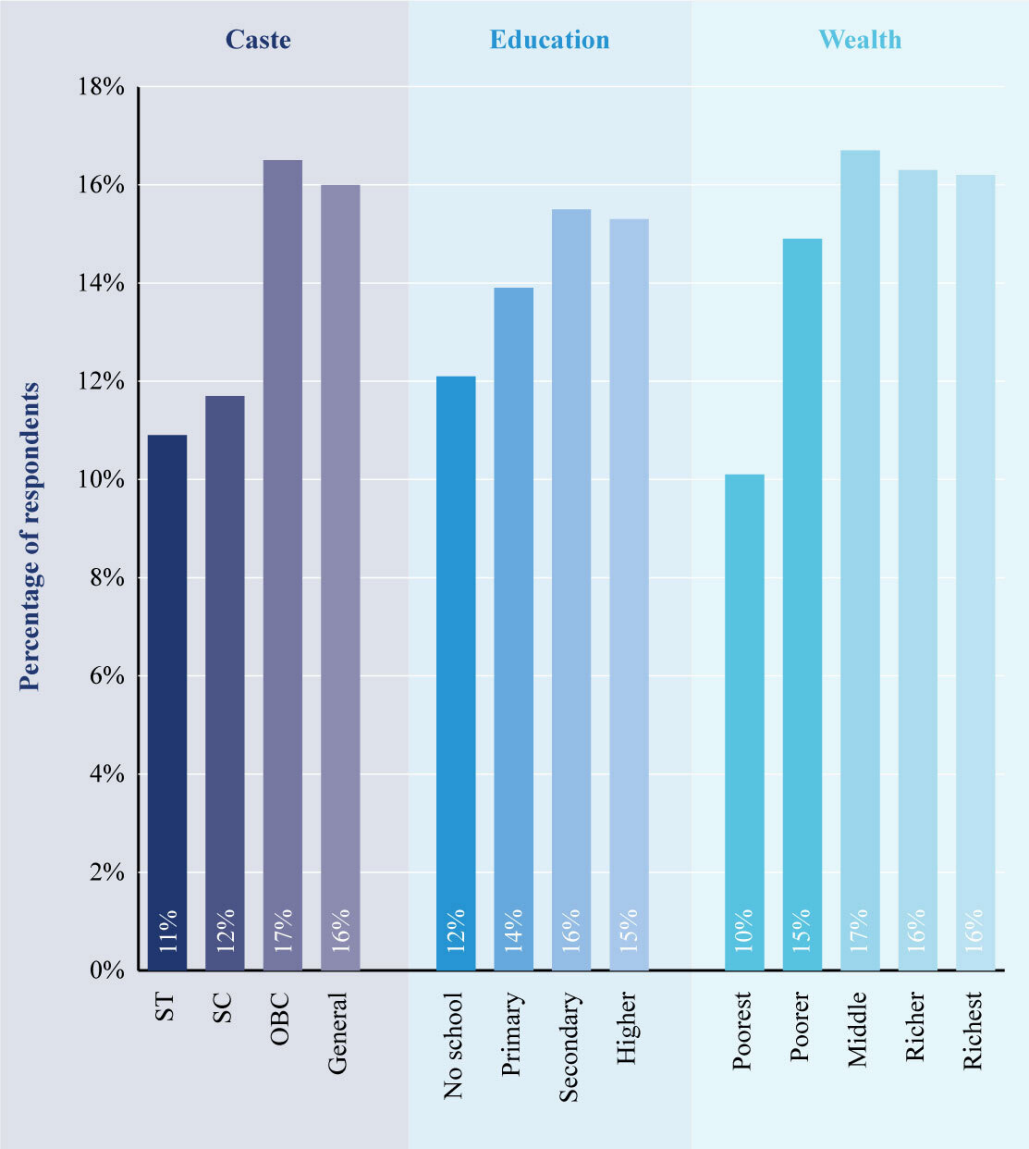
Characteristics of subscribers in the intervention arm of randomised controlled trial (RCT) receiving at least 50% of cumulative Kilkari content in the intervention arm of RCT. OBC, other backward classes; SC, scheduled caste; ST, scheduled tribe.

Overall, those in the most marginalised groups in the intervention arm of the Kilkari RCT sample—that is, those in scheduled castes and ST, those with no education and the poorest, were less likely to be exposed to Kilkari (figure 3). This could be due to factors including more frequent changes in mobile phone numbers (SIM churn) among these groups, and these groups being less able to answer calls during the day, less able to maintain the battery charge of their phones, less able to retain sufficient phone credit to receive calls and more likely to live in areas with poor network connectivity. While no subscribers were deactivated by the RCT for low listening or not answering calls, in the scaled Kilkari programme in 13 states, SIM churn could result in subscribers not receiving calls and, hence, being deactivated to free up infrastructure for Kilkari subscribers who were able to answer calls. This could result in marginalised subgroups being more likely to be deactivated by the scaled programme, while those staying on until the end of the programme might be those with better access to phones and more reliable network connectivity.

## Who is affected by Kilkari?

Programmes may affect healthy behaviours, but do so inequitably; Evidence from other health (non-digital) interventions in maternal, newborn and child health indicate that the greatest benefits have accrued to the population at the top of the socioeconomic scale while the marginalised gain fewer benefits.^34^ By design, ‘direct to beneficiary’ mobile health communication programmes can only achieve impact among those with access to a mobile phone. This means an analysis of who is left behind by such interventions and how to reach those groups through alternative communication channels and platforms should be undertaken at the intervention design phase to set reasonable expectations of impact.

In addition, considerable heterogeneity exists in the way different sub-groups enrolled in a digital ‘direct to beneficiary’ health communication programme interact with the intervention and respond to it. This necessitates an analysis of the way different subgroups interact and respond to the intervention among those who are eligible and subscribed to it.

**Figure 4:**
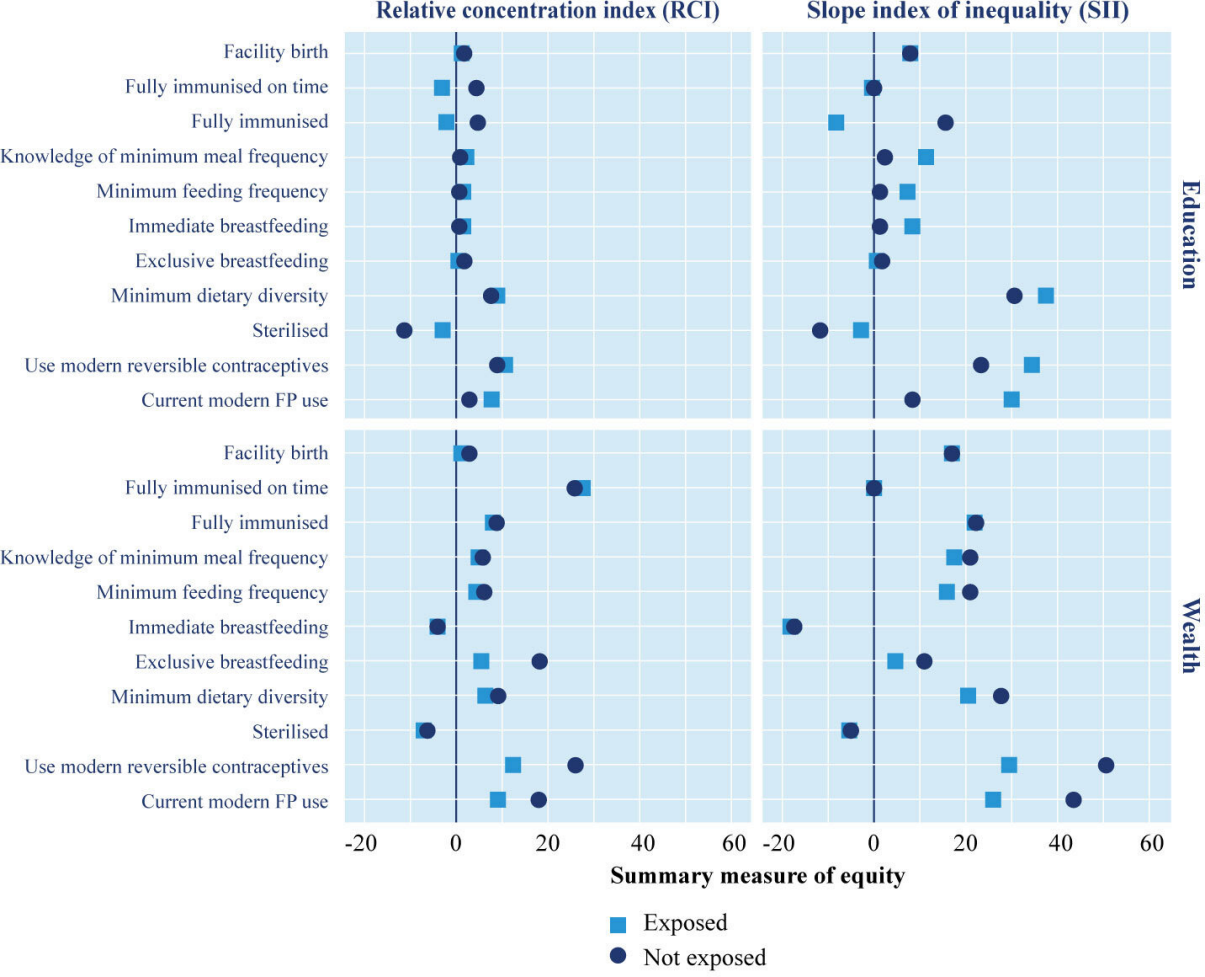
Summary measures of equity for different health behaviours between subscribers who were exposed and not exposed to Kilkari in the intervention arm of the RCT. RCT, randomised controlled trial.

Figure 4 presents the Slope of Index Inequality (SII) and Relative Concentration Index (RCI) for different health behaviours between those exposed and not exposed to Kilkari in the intervention arm of the RCT. The RCI depicts the relative disparity in the levels for a range of RMNCH outcomes (health indicators) while the SII depicts the absolute disparity. The magnitude of both relative and absolute summary measures is related to the overall prevalence of the health behaviours. Relative measures like RCI, tend to be larger at lower prevalence levels while absolute measures, like the SII, tend to be low at both very low and very high prevalence levels.^35^In figure 4, the vertical line represents 0 (no inequity); if the values are positive (to the right of the vertical line) then the health outcome is concentrated in the ‘better off’ and if the values are negative (to the left of the vertical line) then the health outcome is concentrated in the ‘worse off’.

There are three key takeaways from the analysis of the summary measures of equity:

### Not all health behaviours are similar in their equity distribution

Findings from the comparison of summary measures suggest that inequity exists for most health indicators among respondents in the intervention arm of the Kilkari RCT (figure 4). Some health indicators like exclusive breastfeeding show very little difference by socioeconomic position while others like sterilisation are concentrated among the worse off. Contraceptive use (modern methods or reversible) and dietary diversity are the most inequitable—in that they are concentrated among the better off. Such variability in the distribution could be ascribed to other factors like cost of adoption (Poor women may have no access to milk substitutes forcing them to rely on exclusive breastfeeding) or other facilitating factors (Sterilisation is promoted primarily by the front-line health workers in the public sector who tend to focus these efforts on the poor)

### Kilkari appears to close gaps across wealth quintiles more than across education levels

Inequities exist across both wealth quintiles and educational levels for different health behaviours. In general, the magnitude of the levels of inequity is greater across the wealth quintiles than across education levels—that is, the gap between the poorest and richest is wider than the gap between those with no education and higher education—but this pattern is not consistent (eg, immunisation and breastfeeding indicators). Among those exposed to Kilkari when compared with those not exposed, the inequities in wealth (figure 4 - top panels) linked to many health behaviours are lesser than the inequities in education (figure 4 - bottom panels). This is evidenced by the proximity of the pink dots (level of equity among those exposed to Kilkari to the vertical line than the blue dots (level of equity among those not exposed to Kilkari) in the bottom panels compared with the top panels in figure 4. This means that listening to Kilkari moved the needle much more for the population that was ‘worse off’ in terms of wealth but, simultaneously, ‘better off’ in terms of education (poor but at least started secondary education).

### Kilkari appears to allow the ‘worse off’ to make proportional gains in line with the ‘better off’

Among those exposed and not exposed to Kilkari, relative differences (RCI) appear to be smaller than absolute differences (SII)—the blue and pink dots are closer to each other on the left panels than the ones on the right in figure 4. This means that differences in the levels of the health behaviours across wealth quintiles and educational levels between the exposed and not exposed to Kilkari groups were proportional to their actual levels—Kilkari exposure helped ‘lift the boat’ equally (relative to each other) but those ‘better off’ made greater absolute gains due to their pre-existing advantage (figure 4).

**Figure 5:**
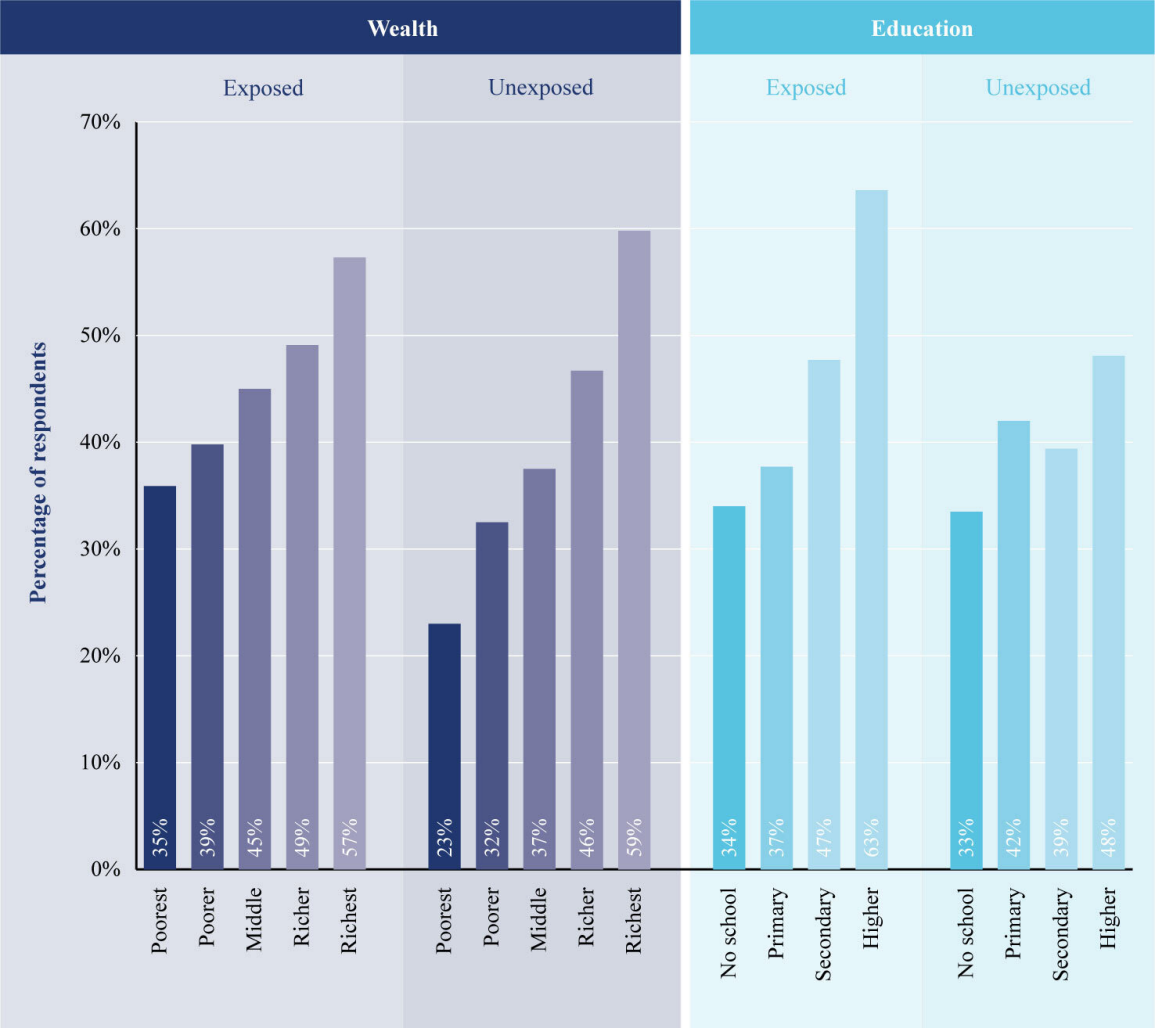
Prevalence of current modern contraceptive use among Kilkari subscribers in the intervention arm of RCT across education levels and wealth quintiles. RCT, randomised controlled trial.

The above points are illustrated in figure 5 using the ‘current modern contraceptive use’ indicator as an example, and the two extreme categories in wealth (richest vs poorest) and education (no school vs higher education). The absolute gap in use of modern contraception between those with no schooling and higher education among the exposed and not exposed to Kilkari groups is 30 and 15 percentage points, respectively. The gap among the poorest and richest quintile among the exposed and not exposed to Kilkari is 21 and 37 percentage points, respectively. The relative disparity between the same categories in education for exposed and not exposed is 1.9 and 1.4, while it is 1.5 and 2.6 for wealth. Exposure to Kilkari increases the absolute differential across education levels by 15 points while decreasing by 16 points for wealth quintiles in absolute terms. In relative terms, this translates to a higher differential across education by a magnitude of 0.5 while that for wealth is lower by a magnitude of 1.1. So, exposure to Kilkari appears to benefit the higher educated among those poorest in terms of wealth quintiles.

The bottom line is that health behaviours show wide variation in the distribution across education and wealth. Digital ‘direct to beneficiary’ communication programmes that seek to improve health through behavioural change communication with the population may have differential impact on those who can understand and act on the information provided. Hence the presence of some level of education (from start of secondary education), by priming the population for receipt of information, appears to act as an effect modifier on the pathway to programme impact. The way forward for digital ‘direct to beneficiary’ communication programmes may be to customise and target health information based on the characteristics of specific segments of the population to help close certain gaps in health behaviours.^36^ This may not be straightforward since it entails collection of subscriber characteristics difficult to measure (wealth) or considered sensitive (ethnicity and social groupings).

## Conclusions

By necessity, ‘direct to beneficiary’ mobile health communication programmes target those with phone access and will thus exclude the poorest and most marginalised. Even among those with access to phones, there is inequity among those who can be reached by mobile health interventions, while changes in health behaviours will vary depending on the subgroups exposed, and the type of health behaviour. In the case of Kilkari, women who did not have access to phones or were not registered in government tracking databases were not subscribed to the intervention. Unless the gender gap in mobile phone access is addressed, inequities in the population coverage and reach of Kilkari will persist. Among those enrolled in Kilkari, the programme’s call retry algorithm bolstered efforts to reach the most marginalised but, overall cumulative exposure to content was still lower among the poorest, least educated and the most disadvantaged castes. Research indicates that exposure to Kilkari has helped improve levels of some but not all health behaviours across marginalised groups.^25^ Education appears to be a key enabler to help improve health behaviours among those exposed to Kilkari content. The study findings also highlight the need for programmes to understand their differential effects on equity through rigorous evaluative methodologies.

## Data Availability

The RCT data are available upon request from Diwakar Mohan (corresponding author) and Amnesty LeFevre (the study PI). NFHS-4 data is available for download from the Demographic and Health Surveys website (https://dhsprogram.com/data/available-datasets.cfm).

## References

[1] Deshmukh M. P M. Addressing Gender and Women’s Empowerment In mHealth for MNCH An Analytical Framework 2013.

[2] Khan NUZ, Rasheed S, Sharmin T, et al. How can mobile phones be used to improve nutrition service delivery in rural Bangladesh? BMC Health Serv Res 2018;18:530. 10.1186/s12913-018-3351-z 29986733 PMC6038298

[3] Njoroge M, Zurovac D, Ogara EAA, et al. Assessing the feasibility of eHealth and mHealth: a systematic review and analysis of initiatives implemented in Kenya. BMC Res Notes 2017;10:90. 10.1186/s13104-017-2416-0 28183341 PMC5301342

[4] Chib A, Lwin MO, Ang J, et al. Midwives and mobiles: using ICTs to improve healthcare in Aceh Besar, Indonesia1. Asian J Commun 2008;18:348–64. 10.1080/01292980802344182

[5] Khan NUZ, Rasheed S, Sharmin T, et al. Experience of using mHealth to link village doctors with physicians: lessons from Chakaria, Bangladesh. BMC Med Inform Decis Mak 2015;15:62. 10.1186/s12911-015-0188-9 26242574 PMC4526289

[6] Ahmed T, Lucas H, Khan AS, et al. eHealth and mHealth initiatives in Bangladesh: a scoping study. BMC Health Serv Res 2014;14:260. 10.1186/1472-6963-14-260 24934164 PMC4072608

[7] Lund S, Hemed M, Nielsen BB, et al. Mobile phones as a health communication tool to improve skilled attendance at delivery in Zanzibar: a cluster-randomised controlled trial. BJOG 2012;119:1256–64. 10.1111/j.1471-0528.2012.03413.x 22805598

[8] Marcolino MS, Oliveira JAQ, D'Agostino M, et al. The impact of mHealth interventions: systematic review of systematic reviews. JMIR Mhealth Uhealth 2018;6:e23. 10.2196/mhealth.8873 29343463 PMC5792697

[9] Alam M, Banwell C, Lokuge K. The effect of women's differential access to messages on their adoption of mobile health services and pregnancy behavior in Bangladesh: retrospective cross-sectional study. JMIR Mhealth Uhealth 2020;8:e17665. 10.2196/17665 32706694 PMC7399959

[10] Alam M, Banwell C, Lokuge K. The effect of women's differential access to messages on their adoption of mobile health services and pregnancy behavior in Bangladesh: retrospective cross-sectional study. JMIR Mhealth Uhealth 2020;8:e17665-e:e17665. 10.2196/17665 32706694 PMC7399959

[11] Barron P, Peter J, LeFevre AE, et al. Mobile health messaging service and helpdesk for South African mothers (MomConnect): history, successes and challenges. BMJ Glob Health 2018;3:e000559. 10.1136/bmjgh-2017-000559 PMC5922496 29713503

[12] Blauvelt C, West M, Maxim L, et al. Scaling up a health and nutrition Hotline in Malawi: the benefits of multisectoral collaboration. BMJ 2018;363:k4590. 10.1136/bmj.k4590 30530659 PMC6282726

[13] LeFevre AE, Shah N, Bashingwa JJH, et al. Does women's mobile phone ownership matter for health? Evidence from 15 countries. BMJ Glob Health 2020;5:e002524. 10.1136/bmjgh-2020-002524 PMC7245424 32424014

[14] Rowntree O, Shanahan M. Connected women: the mobile gender gap report 2020. London, England: GSM Association, 2020.

[15] Khatun F, Heywood AE, Hanifi SMA, et al. Gender differentials in readiness and use of mHealth services in a rural area of Bangladesh. BMC Health Serv Res 2017;17:573. 10.1186/s12913-017-2523-6 28821243 PMC5563057

[16] Mohan D, Bashingwa JJH, Tiffin N, et al. Does having a mobile phone matter? linking phone access among women to health in India: an exploratory analysis of the National family health survey. PLoS One 2020;15:e0236078. 10.1371/journal.pone.0236078 32687527 PMC7371204

[17] Scott K, Ummer O, Shinde A. On behalf of the Kilkari impact evaluation team. another voice in the crowd: the challenge of changing family planning and child feeding practices through mHealth messaging in rural central India. BMJ Global Health. Submitted. 10.1136/bmjgh-2021-005868 PMC8327813 34312156

[18] George AS, Morgan R, Larson E, et al. Gender dynamics in digital health: overcoming blind spots and biases to seize opportunities and responsibilities for transformative health systems. J Public Health 2018;40:ii6–11. 10.1093/pubmed/fdy180 PMC6294040 30307517

[19] Murthy N, Chandrasekharan S, Prakash MP, et al. The impact of an mHealth voice message service (mMitra) on infant care knowledge, and practices among low-income women in India: findings from a Pseudo-Randomized controlled trial. Matern Child Health J 2019;23:1658–69. 10.1007/s10995-019-02805-5 31584144 PMC6823296

[20] Lund S, Nielsen BB, Hemed M, et al. Mobile phones improve antenatal care attendance in Zanzibar: a cluster randomized controlled trial. BMC Pregnancy Childbirth 2014;14:29. 10.1186/1471-2393-14-29 24438517 PMC3898378

[21] Lund S, Rasch V, Hemed M, et al. Mobile phone intervention reduces perinatal mortality in Zanzibar: secondary outcomes of a cluster randomized controlled trial. JMIR Mhealth Uhealth 2014;2:e15. 10.2196/mhealth.2941 25098184 PMC4114456

[22] Murthy N, Chandrasekharan S, Prakash MP, et al. Effects of an mHealth voice message service (mMitra) on maternal health knowledge and practices of low-income women in India: findings from a pseudo-randomized controlled trial. BMC Public Health 2020;20:820. 10.1186/s12889-020-08965-2 32487065 PMC7268375

[23] WHO. Who guideline: recommendations on digital interventions for health system strengthening. Geneva: World Health Organization, 2019. 31162915

[24] Tanahashi T. Health service coverage and its evaluation. Bull World Health Organ 1978;56:295–303. 96953 PMC2395571

[25] LeFevre A, Agarwal S, Chamberlain S, Scott K, et al. Are stage-based health information messages effective and good value for money in improving maternal newborn and child health outcomes in India? protocol for an individually randomized controlled trial. Trials 2019;20:272. 10.1186/s13063-019-3369-5 31092278 PMC6521473

[26] LeFevre A, Agarwal S, Chamberlain S, et al. Are stage-based health information messages effective and good value for money in improving maternal newborn and child health outcomes in India? protocol for an individually randomized controlled trial. Trials 2019;20:272. 10.1186/s13063-019-3369-5 31092278 PMC6521473

[27] Mohan D, Bashingwa JJH, Chamberlain S. Optimising the reach of mobile health messaging programs in India: an analysis of system generated data for the Kilkari program across 13 states BMJ Glob health. Submitted. 10.1136/bmjgh-2022-009395 PMC9366343 35940611

[28] International Institute for Population Sciences - IIPS/India, ICF. India national family health survey NFHS-4 2015-16. Mumbai, India: IIPS and ICF, 2017.

[29] Wulandaria RD, Laksonob AD. Education as predictor of the knowledge of pregnancy danger signs in rural Indonesia. Education 2020;13.

[30] Kumar A, Singh A. Explaining the gap in the use of maternal healthcare services between social groups in India. J Public Health 2015;14:fdv142–81. 10.1093/pubmed/fdv142 28158846

[31] Jennings L, Omoni A, Akerele A, et al. Disparities in mobile phone access and maternal health service utilization in Nigeria: a population-based survey. Int J Med Inform 2015;84:341–8. 10.1016/j.ijmedinf.2015.01.016 25737460

[32] Dutt P, Godfrey A, Chamberlain S, et al. Using behavioural design and theories of change to inform health communication solutions: evolution, evidence and learnings from practice. Family Medicine and Community Health. 10.1136/ihj-2022-000139 PMC10241028 37440851

[33] Chamberlain S. Ten lessons from scaling and transitioning one of the largest mHealth communication programmes in the world to a national government. BMJ Glob Health. 10.1136/bmjgh-2021-005341 PMC8728354 34312151

[34] Barros AJD, Ronsmans C, Axelson H, et al. Equity in maternal, newborn, and child health interventions in countdown to 2015: a retrospective review of survey data from 54 countries. Lancet 2012;379:1225–33. 10.1016/S0140-6736(12)60113-5 22464386

[35] Houweling TA, Kunst AE, Huisman M, et al. Using relative and absolute measures for monitoring health inequalities: experiences from cross-national analyses on maternal and child health. Int J Equity Health 2007;6:15. 10.1186/1475-9276-6-15 17967166 PMC2173893

[36] Pérez-Lu JE, Bayer AM, Iguiñiz-Romero R. Information = equity? how increased access to information can enhance equity and improve health outcomes for pregnant women in Peru. J Public Health 2018;40:ii64–73. 10.1093/pubmed/fdy177 PMC6294033 30307537

[37] Vyas S, Kumaranayake L. Constructing socio-economic status indices: how to use principal components analysis. Health Policy Plan 2006;21:459–68. 10.1093/heapol/czl029 17030551

[38] Team RC. R: A language and environment for statistical computing. In: Computing RFfS. Vienna, Austria, 2019.

[39] Organization WH. Health Equity Assessment Toolkit Plus (HEAT Plus). In: Software for exploring and comparing health inequalities in countries. Upload database edition. Version 1.0, 2017.

[40] Hosseinpoor AR, Schlotheuber A, Nambiar D, et al. Health equity assessment toolkit plus (heat plus): software for exploring and comparing health inequalities using uploaded datasets. Glob Health Action 2018;11:20–30. 10.1080/16549716.2018.1440783 PMC6041818 29974823

[41] Hosseinpoor AR, Nambiar D, Schlotheuber A, et al. Health equity assessment toolkit (heat): software for exploring and comparing health inequalities in countries. BMC Med Res Methodol 2016;16:141. 10.1186/s12874-016-0229-9 27760520 PMC5069829

[42] Hosseinpoor AR, Bergen N, Barros AJD, et al. Monitoring subnational regional inequalities in health: measurement approaches and challenges. Int J Equity Health 2016;15:18. 10.1186/s12939-016-0307-y 26822991 PMC4730638

[43] Wagstaff A, Paci P, van Doorslaer E. On the measurement of inequalities in health. Soc Sci Med 1991;33:545–57. 10.1016/0277-9536(91)90212-U 1962226

